# The Disqualification of Marriage

**Published:** 1921-11

**Authors:** 


*To the Editor of* The Hospital and Health Review.

### The Disqualification of Marriage.

*To the Editor of* The Hospital and Health Review.

Sir,—Has your attention been called to the case of a Maternity and Child Welfare Lady Doctor who has been dismissed from her post because she married? If so cannot something be done about this? It seems very hard on her and if we are all to be treated like this it is a great hardship. There are many married women in business and in the various professions and they are not unfit for their work because of marriage. Of course, we all know how much people like to interfere with other people's business, and I suppose that is the reason for the dismissal of this doctor.—Yours faithfully,

"Staff Nurse."

[We have dealt with this subject in Notes and Comments.—Editor, H. & H.R.]

